# Anterior ischemic optic neuropathy in a case of Takayasu’s arteritis

**DOI:** 10.4103/0974-620X.64236

**Published:** 2010

**Authors:** Suneetha Nithyanandam, Ajoy Mohan, Urmi Sheth

**Affiliations:** Department of Ophthalmology, St Johns Medical College Hospital, Bangalore, India

Ocular involvement in Takayasu’s arteritis (TA) is common and occurs in up to 45%.[1,2] of cases. The most common manifestation is Takayasu’s retinopathy (TR). Four stages of TR are described and they include stage 1 dilatation of small vessels; stage 2 capillary microaneurysm formation; stage 3 arteriovenous anastomoses; and stage 4, further ocular complications including ocular ischemic syndrome.[2] Anterior ischemic optic neuropathy (AION) is a rare manifestation of TA.[2–5] We report a patient with TA who developed AION in the right eye (OD). The patient had lost vision in her left eye (OS) a year earlier, as gathered from medical records, due to central retinal artery occlusion (CRAO).

A 30-year-old female patient presented with acute loss of vision OD of one days duration, which was preceded by transient visual obscurations and syncopal attacks since three months. She was on irregular long term steroid therapy for Takayasu’s arteritis since four years, which she had abruptly stopped 20 days prior to the onset of symptoms. Her previous medical records showed complete loss of vision OS due to occlusion of the central retinal artery 1 year earlier.

The patient was of average build and poor nutrition with absent peripheral pulses and non-recordable blood pressure in both upper limbs. The carotid pulses were not palpable bilaterally. Blood pressure recorded in the lower limb was 140/84 with normal peripheral pulse.

**Figure 1:**
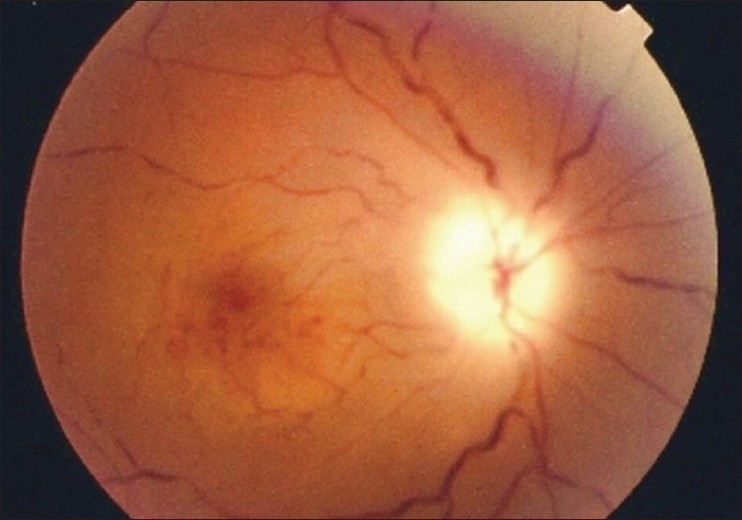
Fundus photograph of right eye showing pallid edema of the optic disc with retinal whitening. The arteries are attenuated, blood column in the veins show segmentation and microaneurysms are seen

**Figure 2:**
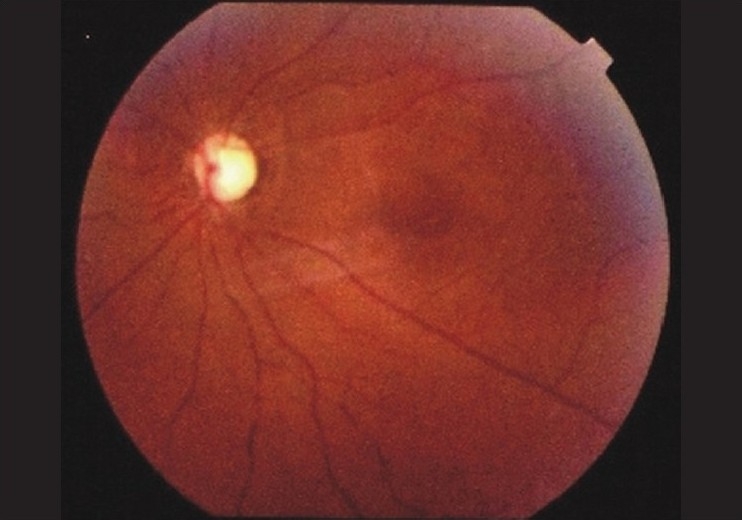
Fundus photograph of left eye showing optic disc pallor with attenuation of the retinal blood vessels; findings suggestive of old central retinal artery occlusion

Ocular examination revealed a best corrected visual acuity of 20/400 OD and absent light perception OS, with bilateral afferent pupillary defect. Anterior segment examination was normal. Intraocular pressures were 8 mmHg OD and 10 mmHg OS. Fundoscopy revealed pallid disc edema with diffuse retinal whitening, severely attenuated arteries, segmentation of the blood column in the veins and microaneursym formation OD [Figure 1], and total optic atrophy with attenuated arteries and absent foveal reflex OS [Figure 2].

Therapy was initiated with a pulse of intravenous methylprednisolone 1gm/day for three days. The patient then received a slow taper of oral prednisolone over weeks to a low maintenance dose of 10 mg/day. Visual acuity improved to 20/200 a week after starting steroids along with a decrease in the number of syncopal attacks. The patient was lost to follow-up after three months.

TA is an idiopathic obstructive arteritis of large and medium sized arteries with a predilection for the aorta and its major branches. This patient fulfils four of the six criteria described by the American College of Rheumatology.[6] It includes patients age being less than 40 years, bilaterally absent brachial pulse, difference in the blood pressures greater than 10 mmHg between the arms and lower limbs and angiographic evidence of obstruction of the large arteries of the upper limbs and the carotids.

Ocular involvement usually occurs due to ocular hypoperfusion which results in ocular ischemic syndrome and TR. TA may also cause hypertension which may lead to hypertensive retinopathy, but this is infrequently encountered.[5] AION is an uncommon manifestation of TA with most of the patients being young.[2–5] AION is attributed to progressive narrowing of major vessels of the neck and resultant ocular hypoperfusion. Hypo-perfusion can at times progress to CRAO as was seen in this patient. However it is possible that the visual loss in OS was also due to ischemic optic neuropathy. The retinal arterioles and retinal background do not look as attenuated and atrophic as usually seen post CRAO. Possibly this is a sequential bilateral AION and this has been reported in literature.[3]

Occurrence of AION in our patient may have been precipitated by abrupt cessation of steroids causing rebound inflammation. Education of the patient and caregivers is essential to mitigate the catastrophic ocular complications of TA seen in our patient. Patient compliance in adhering to treatment protocol and follow-up is mandatory for a favorable outcome.
